# Experiences of Individuals with Cutaneous Leishmaniasis Receiving Intralesional Sodium Stibogluconate or Liquid Nitrogen Cryotherapy in Addis Ababa, Ethiopia—A Cross-Sectional Study

**DOI:** 10.3390/tropicalmed10080203

**Published:** 2025-07-23

**Authors:** Mirna S. Abd El Aziz, Shimelis N. Doni, Edelawit L. Dereje, Petros H. Gebre, Hanna B. Temesgen, Yeabsera W. Zegeye, Saba M. Lambert, Stephen L. Walker

**Affiliations:** 1 Faculty of Infectious and Tropical Diseases, London School of Hygiene and Tropical Medicine, London WC1E 7HT, UK; 2 ALERT Comprehensive Specialized Hospital, Addis Ababa 4010, Ethiopiahannatemesgen3@gmail.com (H.B.T.);

**Keywords:** localised cutaneous leishmaniasis (LCL), SSG, local therapy, pain, neglected tropical disease, *L. aethiopica*

## Abstract

Localised cutaneous leishmaniasis (LCL) is a common neglected tropical disease in Ethiopia, which is mainly treated with intralesional (IL) pentavalent antimonial such as sodium stibogluconate (SSG) and/or cryotherapy. Both treatments are painful, and studies are lacking on the pain associated with these or affected individuals’ experiences of them. A cross-sectional, observational study was conducted at ALERT Comprehensive Specialized Hospital, Addis Ababa/Ethiopia. The socio-demographic and clinical data of individuals affected by LCL receiving IL SSG and/or cryotherapy was gathered, and their treatment was observed. Participants quantified their treatment-associated pain using the Wong–Baker Pain Scale. Health-related quality of life was measured using the (Children’s) Dermatology Life Quality Index. Adverse effects, participant experiences with local therapies, and dermatologists’ experiences and opinions of local LCL treatment were assessed using structured questionnaires. Of the thirty-six individuals with LCL included (64% male, 14% children), 52% reported a treatment-associated pain score ≥ 8. Cryotherapy administered with a cotton bud was associated with lower pain scores ≤ 6 (odds ratio: 0.15, 95% confidence interval: 0.03–0.89) compared to a cryotherapy spray device. There was wide variation in treatment administration. Local LCL treatment is painful, and most individuals experience significant pain. This study highlights the need for less painful but effective treatments, structured training, and clear standard operating procedures.

## 1. Introduction

Leishmaniasis is a vector-borne neglected tropical disease caused by *Leishmania* species transmitted by sandfly bites [1]. Cutaneous leishmaniasis (CL) is common in Ethiopia, with an estimated incidence of 20,000–50,000 cases per year, and is mainly caused by *Leishmania aethiopica* [2,3]. It can present as localised CL (LCL), mucocutaneous leishmaniasis (MCL), or diffuse CL (DCL) [4]. LCL is confined to the skin and develops at the site of the sandfly bite, mostly on exposed body parts such as the face or extremities [5].

Skin lesions caused by *L. aethiopica* may heal spontaneously, but they may also take years and can cause scarring, leading to visible anatomical changes and functional impairment [2,6,7]. CL is treated with either local or systemic (oral or parenteral) therapies determined by factors such as the type of CL, the size, number, and location of lesions, the preference of the affected individual and treating physician, and treatment availability [6,8].

Local therapies include intralesional (IL) pentavalent antimonial (sodium stibogluconate [SSG] or meglumine antimoniate [MA]), cryotherapy, thermotherapy, laser therapy, photodynamic therapy, and 15% paromomycin ointment [8]. Systemic treatment options include SSG and MA, amphotericin B, miltefosine, paromomycin, and pentamidine isethionate [8,9]. There are few data on the efficacy or effectiveness of treatments for CL caused by *L. aethiopica* [6,10].

In Ethiopia, IL SSG and liquid nitrogen (LN) cryotherapy are the two most frequently used local treatments for LCL, either alone or in combination [2,11]. While SSG is provided free of charge in Ethiopia, individuals need to pay for cryotherapy treatment. LN (−196 °C) can be applied using either a cryotherapy spray device or a cotton bud. *Leishmania* parasites in the skin are killed by an immune response induced by the cold [8,12,13,14]. Adverse effects (AEs) include erythema, local oedema, blistering, pain, and pigmentary changes [1,12,13]. The mechanism of pentavalent antimonial action is not fully understood and includes damaging *Leishmania* DNA, causing apoptosis [15,16]. An advantage of IL application is the avoidance of systemic AEs such as cardiotoxicity, myalgia, and arthralgia [17,18]. IL therapy-induced AEs include injection-site oedema and pain, erythema, pruritus, and secondary bacterial infection [18,19].

The guidance on the use of LN cryotherapy and IL pentavalent antimonial varies. Neither the interval between applications of successive treatment, the duration of the application of the cryogen or the number of separate freezes, nor the number of injections or the volume of pentavalent antimonial, has been established [9,20,21,22,23].

Cryotherapy and IL SSG are painful treatments. A recent study from Ethiopia on the effectiveness of IL SSG for individuals with LCL reported that 95.8% of participants receiving IL SSG experienced injection-site pain, which was reported as mild in 81.9% [19]. The authors did not specify how the pain was assessed.

There are no published studies that have quantified individuals’ pain associated with local treatment for CL. Pain can have an important impact on individuals’ treatment experience and adherence. We aimed to measure the pain associated with cryotherapy and/or IL SSG in individuals with LCL. We wished to explore the experiences of individuals affected by LCL treated with either or both modalities.

## 2. Materials and Methods

The study was conducted at the Dermatovenerology outpatient department (OPD) at the ALERT Comprehensive Specialized Hospital (ALERT) in Addis Ababa, Ethiopia, using a cross-sectional, observational, descriptive design, between 11 July and 9 August 2024.

Individuals affected by LCL and dermatologists in training were included as participants. LCL was defined as CL with ≤10 cutaneous lesions, which aligns with a previous Ethiopian clinical research study [24]. Individuals with MCL, who often receive systemic and local treatment at the same time at ALERT, were excluded, to not introduce bias, since the focus was on local treatment. MCL was defined as CL with ≤10 cutaneous lesions, involving the skin and the neighbouring mucosal surfaces [24]. Individuals with clinically confirmed LCL receiving IL SSG and/or LN cryotherapy who were ≥8 years of age and agreed to participate were eligible. Treatment was determined by the responsible clinician. Exclusion criteria included refusal to participate or treatment other than IL SSG and/or LN cryotherapy. All dermatologists in training working in the OPD were invited to participate. Those who agreed provided written consent.

The study was divided into the following five parts: (1) the collection of clinical data using a case report form, (2) assessment of the health-related quality of life associated with LCL, (3) the observation of the treatment procedure, (4) the assessment of pain experienced during treatment, and (5) a participant questionnaire about experiences with local treatment. Additionally, experienced dermatology trainees completed a questionnaire about their experiences with LCL local therapy.

After obtaining informed consent, participants ≥ 16 years of age completed the Amharic version of the Dermatology Life Quality Index (DLQI), and those < 16 years completed the Children’s DLQI (CDLQI) [25,26,27].

Demographic and clinical data were collected, including the number, size, site, and morphology of the lesion(s). The morphology of the lesion was documented by either one of the dermatology residents who were part of the study team (E.L.D., P.H.G., H.B.T., or Y.W.Z.) or the responsible dermatology resident in OPD on the study day. Participants were categorised as “treatment-naïve” if they had not received any treatment with IL SSG and/or cryotherapy in the six months prior to enrolment.

A cryotherapy instruction sheet in the treatment room recommended a freeze duration of 30–60 s, 1–2 freeze/thaw cycles, and 3–5 treatment sessions every 1–2 weeks for the treatment of CL. The treatment administered to each participant was observed by M.S.AEA. using a standardised checklist based on guidelines and recommendations for the application of cryotherapy and IL SSG (File S1A) [21,28,29,30]. Information was gathered on the treatment administered, the technique used, the treating dermatologist’s experience, and the preparation and explanation of the procedure. The number of individual freezes for cryotherapy and the duration of each freeze, as well as the number of separate injections with IL SSG and the volume of antimonial administered, were documented. The timing of each step of the procedure was noted.

Participants were asked to quantify the pain experienced during treatment using the “Wong–Baker FACES Pain Scale” (WBFPS) within 60 min of the procedure. The WBFPS is a numerical scale consisting of six faces that represent different degrees of pain (from 0: “No Hurt” to 10: “Hurts worst”) [31,32]. Previous studies have shown that a WBFPS score of eight or ten translates into a median of 74 or 95 mm on the visual analogue scale (equalling moderate and severe pain), so a score of <8 vs. ≥8 was used for the correlation of pain and individual or healthcare-related factors [33,34].

Participants’ experiences with IL SSG and/or cryotherapy were explored using a standardised questionnaire about AEs, pain, and anxiety compared to the previous treatment session. The questionnaire was developed by M.S.AEA. and S.L.W. for this study. Participants were asked to compare the pain experienced to their expectations and the previous treatment session using a Likert scale (File S1B).

Dermatology trainees who administered treatment independently answered a standardised questionnaire about their experiences with local therapies (File S1C).

Anonymised data were entered into RedCap [35]. Data analysis was performed with STATA/SE 18.0 (StataCorp LLC). Descriptive statistics using proportions, medians, ranges and standard deviations, and potential correlations were explored by cross-tabulating subgroups with reported pain scores and other outcome variables. Unadjusted odds ratios were calculated between groups with a “high” WBFPS pain score (≥8) and lower scores (≤6).

Ethical approval was obtained from the Research Ethics Committee of the London School of Hygiene and Tropical Medicine and the AHRI/ALERT Ethics Committee. Participants with LCL who were under 18 years of age provided assent, and their responsible adult consented. When a written signature was not possible, participants could consent with a thumb print witnessed by an adult, independent of the study, who signed to confirm consent.

## 3. Results

**Figure 1:**
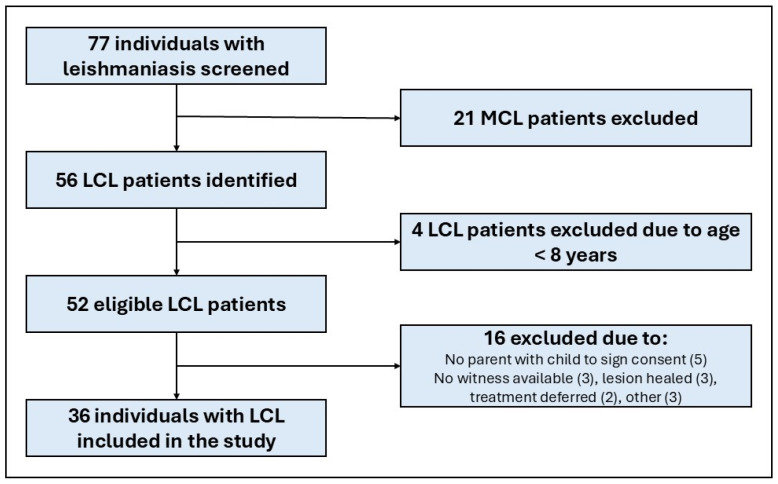
Flow chart of participant identification, exclusion, and inclusion. LCL, localised cutaneous leishmaniasis; MCL, mucocutaneous leishmaniasis.

In total, 77 individuals with CL (43 [56%] male, 18 [23.4] children) attended the dermatology OPD during the study period. Thirty-six individuals with LCL were included in the study (Figure 1).

Table 1 shows the demographic and clinical data of the participants with LCL.

Thirty-one adult participants completed the DLQI. The median score was six (range: 1–24). Five children completed the CDLQI, with a median score of 5 (range: 2–17).

### 3.1. Treatment Observation

Fifteen dermatology trainees (12 [80%] female) were observed administering local treatments for LCL. Eight treatments were administered by first-year trainees, of whom four were supervised by an experienced second- or third-year trainee.

Twelve (80%) were experienced in administering local treatment independently and were interviewed about their experiences and opinions of LCL therapy.

Table 2 shows an overview of the treatments that were observed to be administered. In total, 22 treatments with IL SSG and 33 with cryotherapy were observed. Two (6%) participants received their first treatment session on the study day. On the study day, 53% received combination therapy with IL SSG and LN cryotherapy.

#### 3.1.1. Procedure Preparation

No running water was available in the treatment room, so dermatology trainees were unable to wash their hands before treatment application. On twenty-seven occasions (75%), trainees wore gloves. On nine (33%) occasions, this was to apply cryotherapy alone; once, it was (4%) for IL SSG monotherapy; and seventeen (63%) times, it was for combination therapy. Data for three treatment applications were not recorded. Antiseptic (70% alcohol) was not readily available to clean the skin prior to treatment. Only one resident cleaned the lesion and surrounding skin before applying IL SSG.

#### 3.1.2. Treatment Application

Thirty-three individuals received LN cryotherapy. LN was administered to 19 individuals (58%) using a hand-held cryogen spray device (Brymill Cry-Ac^®^ B-700, Brymill, Cannock, UK) and to 14 individuals (42%) using a cotton bud that had been immersed in LN. In total, 11 (33%) participants received one freeze, and 13 (39%) received two freezes. The largest number of observed freezes was eight, for a participant with five lesions. The mean total duration of LN application was 49.5 s (standard deviation [SD] ± 28.3), with a range between 15 s and 132 s. None of the dermatology trainees used a device to time the duration of the freezes.

IL SSG (100 mg/mL) was administered using a 1 mL insulin syringe (30 G × 1/4″, 0.3 × 0.6 mm^2^).

The median number of separate injections with IL SSG that participants received during the treatment session was four (range: 2–17). The volume of SSG infiltrated per procedure ranged from 0.1 to 0.8 mL, with a mean volume of 0.4 mL (SD ± 0.2 mL).

### 3.2. Pain Assessment

Thirty-five participants (97%) reported the pain experienced during the observed treatment using the WBFPS. Eighteen (51%) reported a pain score of eight or ten (“Hurts Whole Lot” and “Hurts Worst”), four (80%) of the children and fourteen (47%) of the adult participants. None of the participants who answered the questionnaire chose “No hurt” (Table 3).

The odds of recipients of LN applied with a spray device reporting a high pain score was 6.5 times the odds of participants receiving LN applied with a cotton bud reporting a high pain score (95% confidence interval: 1.1–37.4, Table S1). There were no other variables significantly associated with a high WBFPS score of ≥8.

### 3.3. Individuals’ Experiences of Local Therapies

Twenty-four (67%) individuals had received local treatment in the department two weeks before participating in the study, and seven (19%) had received it four weeks prior. The other three participants were enrolled three, seven, and twelve weeks after their last treatment. Only 14 (39%) participants reported that they had had the treatment explained to them by a doctor. All 14 stated that they had understood the doctor’s explanation of the procedure.

In total, 31 (91%) of 34 participants with prior experience of cryotherapy and 21 (95%) of 22 individuals who had previously been treated with IL SSG reported associated AEs (Table 4). The most common AE was pain at the application site (88% for cryotherapy and 95% for IL SSG).

One participant (4%) reported that they did not experience any AEs. Data were missing for two participants.

Seventeen individuals with LCL (47%) reported the pain experienced during treatment on the study day to have been more or much more than expected. Nine (25%) found the pain to be as expected, and ten (28%) found it to be less or much less than expected.

Fifteen (44%) participants stated that the pain experienced during treatment application on the study day was much more or more severe compared to the previous treatment session. Thirteen (38%) found the pain less or much less severe.

No participant had taken analgesia before treatment administration.

The majority (18 [53%]) of participants reported being “nervous” during the observed treatment session as during the previous one. Five (15%) were more nervous, and eleven (32%) were less or much less “nervous” than before.

Five participants (14%) felt “relaxed” about their next treatment session, six (17%) felt “nervous”, and 25 (69%) felt neither “relaxed” nor “nervous”. Of those who reported being “nervous”, five (83%) feared the pain caused by treatment. Two (33%) were “nervous” about the costs of being treated, and four (67%) were nervous about returning to the hospital.

### 3.4. Dermatology Trainees’ Experiences and Opinions Regarding Local Therapies

#### 3.4.1. Training and Experience in Administering Local Treatment

All 12 second- and third-year dermatology trainees included in the study had at least six months of experience administering IL SSG or cryotherapy independently. All trainees had been trained in the techniques by observing experienced doctors. Eleven (92%) and ten (83%) had applied IL SSG and cryotherapy under the supervision of an experienced doctor. One trainee had attended didactic teaching for cryotherapy.

#### 3.4.2. Trainees’ Responses Regarding Treatment Application

Ten (83%) trainees reported timing the duration of application of LN cryotherapy and described a variety of methods: “counting the seconds” (*n* = 6), “using a mobile phone/watch” (*n* = 1), and applying cryotherapy in pulses of one second duration (*n* = 1). Respondents stated the CL freeze duration they considered optimal, with answers between 15 s and 60 s.

All trainees agreed that the size of the lesion was critical in determining the volume of IL SSG to administer. However, the assessment of the size differed. Seven (58%) reported assessing the maximum diameter, and four (33%) reported assessing the area of the lesion. Seven (58%) stated that the assessment of the lesion size was an estimation, while the other five did not specify how they measured it.

Trainees reported that they administered 0.1–0.5 mL SSG per cm lesion. Injections were performed at intervals of 1 cm. Seven (58%) trainees stated they used the maximum diameter of the lesion for their calculation of the volume needed, four (33%) stated that they used the area (in cm^2^), and one did not specify a method.

#### 3.4.3. Trainees’ Opinions Concerning Decreased Tolerability to Local Therapies

All 12 trainees believed that very young or older individuals were less likely to tolerate IL SSG and LN cryotherapy. Ten trainees (83%) named the <5-year-olds as the least tolerant age group, and 25–33% named the 5–10-year age group and people > 60 years old. All nine (75%) trainees who mentioned sex as a relevant factor reported that female individuals tolerated IL treatment less well than male ones. For cryotherapy, the duration of the freeze, the number of freeze/thaw cycles, and sex were believed to be important factors by ten (83%), nine (75%), and eight (72%) trainees, respectively. Concerning the tolerability of IL SSG, all trainees thought that the number of separate injections led to decreased tolerability, while 9 (75%) and 7 (58%) named the amount of volume injected and previous injections as relevant. Two trainees mentioned that a lack of information on the procedure can lead to decreased tolerability. Three (25%) believed that certain lesion sites, especially facial lesions or lesions involving the cartilage of nose or ear, reduced tolerability.

Eleven (21%) trainees stated that there was a difference between monotherapy with cryotherapy or IL SSG and combination therapy with respect to individuals’ treatment tolerability. Seven (55%) reported that combination therapy was more painful, and five (45%) reported that it was more “stressful” due to the longer procedure duration. Two (18%) believed that combination therapy was less painful.

#### 3.4.4. Trainees’ Opinions on Factors Associated with Treatment Discontinuation

The distance to the hospital was seen as the most important factor in the risk of treatment discontinuation, followed by treatment costs. Six (50%) trainees believed that pain due to cryotherapy was a very important or important factor in discontinuation, whereas only one trainee found this to be the case for IL SSG. The other trainees felt that it was only a moderately or slightly important factor.

## 4. Discussion

Our findings show that the treatment of CL with LN cryotherapy or IL SSG is painful, with 100% of participants reporting that they experienced pain during the treatment session. Of the participants, 52% had a pain score of ≥8 on the WBFPS, and amongst them were four of the five children included in the study. The level of pain reported by participants in our study is higher than that reported by Zewdu et al., where 82% experienced “mild” injection-site pain with IL SSG [19]. There are several important differences between the two studies. Our study included individuals receiving cryotherapy or IL SSG or both. We used a tool (previously employed to assess pain in patients with leprosy reactions in Ethiopia) to assess pain shortly after the completion of treatment [36]. It is unclear at what time Zewdu and colleagues assessed AEs in their study.

In our small study, the only factor associated with a WBFPS score of ≥8 was the mode of cryotherapy application. Participants who received cryotherapy with a spray device reported higher pain scores than those who had it applied with a cotton bud. A study from Sri Lanka using the cotton bud application of LN to treat CL reported that the treatment was “very painful”, with the pain lasting for 15–30 min [37]. Further research exploring pain experienced with different forms of cryotherapy application is necessary.

The other common AEs reported by participants receiving cryotherapy in this study were bacterial infection (47%) and pigmentary changes (41%). Similar AEs, but not their prevalence, have been described in previous studies from Ethiopia [38,39]. The most reported AEs associated with IL SSG are injection-site swelling (57%) and bacterial infection (44%). In individuals who have received both treatments, it is not always possible to determine which treatment contributed to which AE. The results of the health-related quality of life assessment in our study were consistent with reported DLQI scores from a recent study on individuals with CL at ALERT [40].

The duration of LN application was very variable. The Ethiopian national guidelines for leishmaniasis highlight that evidence is lacking regarding both the duration and frequency of LN [11]. The Infectious Diseases Society of America and the American Society of Tropical Medicine and Hygiene guidelines state that “multiple regimens” exist and give an example of a freeze duration of 15–20 s [20]. The World Health Organization (WHO) guidelines recommend “blanching of the lesion for 10 s” [9]. Guidance on cryotherapy for other skin conditions such as viral warts and actinic keratosis shows a similar variation in the recommended freeze duration [41,42,43,44]. In a clinical study, the efficacy of LN in the treatment of viral warts was not affected by the application modality (spray device or cotton bud), although the duration of the application was not reported [45]. Further research needs to elucidate the optimal duration of cryogen application for CL.

Participants in our study received a variety of volumes of IL SSG. While the maximum volume administered was 0.8 mL, the Pan American Health Organization Guidelines recommend up to a maximum total volume of 15 mL IL pentavalent antimonial per day [22]. IL antimonial is recommended for individuals with 1–3 lesions of ≤3 cm in diameter, and each lesion should be treated with 3–5 infiltrations of 1–5 mL antimonial. The administered volumes observed in our study were much lower, and the number of injections was often higher. Lesions of >3 cm diameter were treated with IL SSG; the largest one had a maximum diameter of 13 cm.

It is unclear why there is such a large variation in practice with respect to the administration of local treatments for CL. Some possible causes might include the level of experience of the clinician and time constraints. The choice of treatment given is often dependent on availability (e.g., no LN was available on the first study day, and the cryogun did not work on some days) and on the financial means of the affected individual (cryotherapy had to be paid for). The treatment decision was made by the clinician responsible for the treatment facility on the day, so it depended on their personal judgement.

Doctors at ALERT face several challenges in administering local treatment for LCL. Resource limitations such as the lack of running water, soap, and disinfectant affect hygiene practices. Time constraints due to the high number of patients may affect the clinician’s performance in lesion assessment and communication. Only 39% of the participants in this study stated that their doctor had explained the treatment to them.

Time constraints may also explain discrepancies between what clinicians said that they did and what they were observed to do with respect to treatment application. Although 83% reported that they timed the freeze duration when applying cryotherapy, and they all stated that they based their calculation of the volume of SSG on the lesion size, no trainee was observed timing the application of cryogen or measuring the lesion, leading to the assumption that it was solely based on estimations.

There are publications and teaching videos available on the use of cryotherapy for common skin conditions like warts and actinic keratosis, but specific guidance on its practical use for CL is sparse [28,46,47]. The WHO manual on the management of CL in the Eastern Mediterranean Region and the LeishMan recommendations include an explanation of the administration of cryotherapy and IL antimonial [21,48]. The Study and Control of Tropical Diseases Program in Colombia explains the application of IL antimonial for CL step-by-step in a video [29]. To guarantee that all treatment is administered in a standardised way, training sessions with an agreed curriculum and assessments and the development of standard operating procedures for local treatments would be required.

Dermatology trainees believed that females affected by CL were less likely to tolerate local therapies, particularly cryotherapy. However, 13 (59%) males reported a WBFPS score of ≥8 (compared to five [38%] female participants). This difference was not significant but suggests that trainees’ subjective views are not supported by objective measures. The reluctance of males undergoing painful treatment to express their pain in front of medical personnel might explain this apparent contradiction. Studies have found pain assessment in men and women to be complex and associated with gender role expectations [49,50]. There are no similar studies on gender-associated perspectives on pain in Ethiopia.

Our study has several limitations. The sample size was small, only two participants were treatment-naïve, and there were few children or older participants. The cross-sectional design did not allow for an analysis of the evolvement of pain over the course of the treatment. Research exploring different forms of treatments regarding both the experience of pain and treatment outcomes would be of interest. Our study was a single-centre study; future studies in different treatment centres could gather more evidence on the variation in treatment administration and its effect on patient experiences. The WBFPS has been used in Ethiopia as part of a multi-national study of erythema nodosum leprosum but has not been validated in Ethiopia [36]. It was not possible to adjust for potential confounders given the small number of participants. Our observations might have affected the clinicians’ behaviour, leading to a Hawthorne effect; however, this seems unlikely to have been significant, given the discrepancy between what residents reported to be optimal practice and what was observed [51].

The pain associated with local therapies for CL demonstrates the need for approaches to reducing pain and identifying effective, less painful treatments. Topical paromomycin has been shown to be efficacious in CL due to *L. major*, but data are needed for *L. aethiopica* [52,53]. Evidence for the use of topical anaesthetic and lidocaine for reducing pain in the local treatment of LCL is currently lacking. However, a study by Usanakornkul et al. on pain relief in keloid treatment showed a significant reduction in needle-stick pain and a decrease in intralesional injection pain when using topical anaesthetics [54]. Further studies exploring the use of topical anaesthetics and lidocaine prior to the application of IL SSG are needed. Standardised protocols for the optimal administration of local treatment, access to the necessary equipment, and agreed training approaches for healthcare professionals administering local treatments may help to improve experiences of treatment and outcomes. This would be facilitated by improved evidence concerning the duration of cryogen application and the volume of IL antimonial for CL due to *L. aethiopica*.

## Figures and Tables

**Table 1:** Demographic and clinical data of individuals affected by localised cutaneous leishmaniasis, *n* = 36.

Variables	Subgroup	Number of Individuals (%)
Male sex		23 (63.9)
Median age (years [range])	26.5 [9–83]	
Age group (years)	8–15	5 (13.9)
	16–29	15 (41.7)
	30–49	10 (27.8)
	≥50	6 (16.7)
Median duration of current lesion (months, [range])	12 [1–120]	
Duration of current lesion (in groups; months) ^a^	1–6	6 (16.7)
	7–12	5 (13.9)
	>12	25 (69.5)
Previous treatment ^b^	None	12 (33.3)
	Traditional ^c,e^	11 (30.6)
	Medical ^d,e^	19 (52.8)
Number of lesions	1	26 (72.2)
	2	5 (13.9)
	3	4 (11.1)
	5	1 (3)
Median size of index lesion (largest diameter; mm [range])	39.5 [12–130]	
Index lesion size groups (largest diameter; mm)	<25	11 (30.6)
	26–50	18 (50)
	>50	7 (19,5)
Morphology of the index lesion ^e^	Plaque	29 (80.6)
	Indurated	27 (75)
	Hyperpigmented	20 (55.6)
	Erythematous	19 (52.8)
	Crusted	11 (30.6)
	Scaly	7 (19.4)
	Depigmented	7 (19.4)
	Hypopigmented	5 (13.9)
	Other	11 (30.5)
Secondary bacterial infection		5 (13.9)

^a^ If a participant had >1 lesion, the duration of the first lesion was documented. ^b^ This refers to treatment received ≥6 months before study enrolment. Some participants had received both traditional and medical treatment. ^c^ Traditional treatment: herbal (*n* = 10, 90.9%), holy water (*n* = 1, 9.1%). ^d^ Medical treatment: cryotherapy (*n* = 4, 21.1%), unspecified topical treatment (*n* = 12, 63.2%), unspecified oral treatment (*n* = 9, 47.4%), unspecified antibiotics (*n* = 1, 5.3%). ^e^ Multiple answers were possible.

**Table 2:** Type of treatment received for localised cutaneous leishmaniasis, *n* = 36.

Variable	Subgroup	Number of Individuals (%)
Type of local therapy received on study day	Cryotherapy monotherapy	14 (38.9)
	IL SSG monotherapy	3 (8.3)
	Combination therapy (cryotherapy and IL SSG)	19 (52.8)
First or subsequent treatment session	First therapy session (therapy-naïve)	2 (5.6)
	Subsequent therapy session (therapy-experienced)	34 (94.4)
Median treatment session number (range)	4 (1–24)	
Treatment session number	1st	2 (5.6)
	2nd	2 (5.6)
	3rd	5 (13.9)
	4th	7 (19.4)
	5th	4 (11.1)
	6th–10th	10 (27.8)
	≥11th	6 (16.7)
Type of local therapy received before	Cryotherapy monotherapy	10 (29.4)
	Combination therapy (cryotherapy and IL SSG)	24 (70.6)
Systemic treatment with SSG ^a^	Never	23 (63.9)
	Before receiving local therapies	10 (27.8)
	Between receiving local therapies	3 (8.3)

IL, intralesional; SSG, sodium stibogluconate. ^a^ intramuscular application of SSG, usually 20 mg/kg/day for 28 days.

**Table 3:** Adults’ and children’s Wong–Baker FACES Pain Scale scores, *n* = 35.

Wong–Baker FACES Pain Scale Score, *n* (%)						
	2 (Hurts Little Bit)	4 (Hurts Little More)	6 (Hurts Even More)	8 (Hurts Whole Lot)	10 (Hurts Worst)	Total
**Age Group**						
Children	0 (0)	0 (0)	1 (20)	1 (20)	3 (60)	5 (100)
Adults ^a^	5 (16.7)	6 (20)	5 (16.7)	8 (26.7)	6 (20)	30 (100)
**Sex**						
Male ^a^	3 (13.6)	3 (13.6)	3 (13.6)	6 (27.3)	7 (31.8)	22 (100)
Female	2 (15.4)	3 (23.1)	3 (23.1)	3 (23.1)	2(15.4)	13 (100)
**Type of treatment received**						
IL SSG monotherapy	1 (33.3)	0 (0)	1 (33.3)	0 (0)	1 (33.3)	3 (100)
Cryotherapy monotherapy ^a^	3 (23.1)	3 (23.1)	4 (30.8)	3 (23.1)	0 (0)	13 (100)
Combination therapy	1 (5.3)	3 (15.8)	1 (5.3)	6 (31.6)	8 (42.1)	19 (100)
**For participants receiving cryotherapy: treatment modality (*n* = 32)**						
Cryotherapy spray device ^a^	1 (5.6)	2 (11.1)	2 (11.1)	7 (38.9)	6 (33.3)	18 (100)
Cotton bud	3 (21.4)	4 (28.6)	3 (21.4)	2 (14.3)	2 (14.3)	14 (100)

^a^ Data is missing for one participant.

**Table 4:** Adverse effects with cryotherapy and intralesional sodium stibogluconate.

Type of Adverse Effect	Cryotherapy (*n* = 34), *n* (%)	Intralesional SSG (*n* = 22), *n* (%)
Any kind of adverse effect	31 (91.2)	21 (95.5)
Pain at application site	30 (88.2)	20 (95.2)
Bacterial infection	16 (47.1)	10 (47.6)
Pigmentary change	14 (41.2)	8 (38.1)
Blistering	11 (32.4)	5 (23.8)
Swelling at application site	5 (14.7)	12 (57.1)
Scar	3 (8.8)	1 (4.8)
Pruritus	1 (2.9)	0 (0)
Allergic reaction/anaphylaxis	0 (0)	0 (0)
Epistaxis	0 (0)	1 (4.8)

SSG, sodium stibogluconate.

## Data Availability

The database for this study can be found in the Supplementary Materials (File S3).

## Supplementary Materials

The following supporting information can be downloaded at https://www.mdpi.com/article/10.3390/tropicalmed10080203/s1, File S1: Data collection sheets, File S2: STROBE Checklist, File S3: Database, Table S1: Risk of Wong–Baker FACES Pain scores ≥ 8 with individual and healthcare-related factors.

## Abbreviations

The following abbreviations are used in this manuscript:

AE	Adverse effect
ALERT	ALERT Comprehensive Specialized Hospital
CDLQI	Children’s Dermatology Life Quality Index
CL	Cutaneous leishmaniasis
DCL	Diffuse cutaneous leishmaniasis
DLQI	Dermatology Life Quality Index
IL	intralesional
LCL	Localised cutaneous leishmaniasis
LN	Liquid nitrogen
MA	Meglumine antimoniate
MCL	Mucocutaneous leishmaniasis
OPD	Outpatient department
SD	Standard deviation
SSG	Sodium stibogluconate
WBFPS	Wong–Baker Faces Pain Scale
WHO	World Health Organization
