# Supplementary material for: Experiences of Individuals with Cutaneous Leishmaniasis Receiving Intralesional Sodium Stibogluconate or Liquid Nitrogen Cryotherapy in Addis Ababa, Ethiopia—A Cross-Sectional Study
Source: Trop Med Infect Dis. 2025 Jul 23;10(8):203. doi: 10.3390/tropicalmed10080203 (PMC12390606; doi:10.3390/tropicalmed10080203)
Supplement: Supplementary file 1 [file tropicalmed-10-00203-s001.zip › File S1_DataCollectionSheets.pdf]

Appendix S1: Data collection sheets

Page

Appendix S1 A: Therapy observation sheet……………………………...2

Appendix S1 B: Participant Survey……………………………………..4

Appendix S1 C: Doctors Survey…………………………………...…7

Participant ID (patient):_____________________ *Doctor ID:_____________________*

Appendix S1 A:
OBSERVATION OF LCL Therapy

Number of treatment session:
☐ 1st ☐ 2nd ☐ 3rd ☐ 4th ☐ 5th ☐ Other: ___________

Treatment given:
☐ Cryotherapy ☐ Intralesional SSG ☐ Both (cryotherapy, followed by intralesional SSG)

☐ Other: ____

	Done	Not Done
**Introduction / Explanation of procedure**		
**Start Time (Patient entering the room):** _______________________		
Welcomes/greets individual		
Introduces self		
**For treatment-experienced participants:**<br>Asks about any adverse reactions after the last treatment		
Explains the process for cryotherapy /or intralesional therapy		
Seeks consent to proceed		
Asks individual if he/she has any questions		
Guides the individual to position comfortably if required		
**Preparation of procedure**		
**Time:** ____		
Washes hands		
Puts on gloves		
Cleans the lesion and surrounding skin		
Dries the lesion and surrounding skin with gauze		
**Application of treatment**		
**Time at first freeze:** _______________________________________		
Applies liquid nitrogen on the <u>lesion</u> and up to 2 mm <u>outside the lesion margin</u>		
Times the freeze		
Allows a complete thaw		
Repeats the freeze		
Times the repeated freeze		
**Number of freezes:** _______________________________________		
**Duration of 1st freeze:** _________________	**Duration of 2nd freeze:** _________________	
**Duration of 3rd freeze:** _________________	**Duration of 4th freeze:** _________________	
**Duration of 5th freeze:** _________________	**Duration of subsequent freezes:** __________	
**Time at end of last freeze:** __________________________________		
Checks the drug for intralesional therapy		
Checks the expiry date of the drug		
Aspirates antimonial from ampoule into syringe aseptically		

Participant ID (patient):_____________________ *Doctor ID:_____________________*

**Time at beginning of first injection: ____________________________**		
**Number of separate injections: ______________________________**		
Inserts the needle 5-10 mm from the edge of the lesion (into intact skin; bevel upwards, angle 30-45°)		
Positions the needle towards the centre of the lesion		
Aspirates with the plunger (to ensure that it is not inside a vessel)		
Infiltrates the medicine slowly		
Gradually withdraws the needle while administering the antimonial		
**Time at end of last injection: ________________________________**		
**Total amount of antimonial given: ____________________________ ml**		
Wound dressing		
**Further explanations / discharge / scheduling of next visit**		
Explains to the individual how to care for and clean the wound		
Informs the individual about possible adverse reactions		
**Adverse reactions mentioned:**<br>Pain<br>Blistering<br>Infection<br>Colour change	**Yes:**<br>**[ ]**<br>**[ ]**<br>**[ ]**<br>**[ ]**	**No:**<br>**[ ]**<br>**[ ]**<br>**[ ]**<br>**[ ]**
Informs the individual about time for next IL-/cryotherapy session		
Schedules the individual for next facility visit		
Asks individual if she/he has any questions		
**End Time (patient walking out): ______________________________**		

Total time: ____
(from greeting the patient to patient walking out)

Total time spent for the procedure of cryotherapy: _________________
(From time of first freeze to last freeze being finished)

Total time spent for the procedure of intralesional therapy: __________
(From time of first injection to last injection being finished)

Cryotherapy done with: ☐ Cryo-Spray ☐ Cotton bud ☐ NA

Procedure stopped prematurely: ☐ Yes ☐ No

If cryotherapy was given <u>after</u> intralesional therapy, or other changes in treatment procedure: Comment (e.g. how many thaw cycles before and after intralesional therapy given):

____
____
____

የተሳታፊው መለያ:______________________
Participant ID

Appendix S1 B:

የተሳታፊዎች ዳሰሳ፡ የቆዳ ሌይሽማንያሲስ ህክምና ተሞክሮዎች - ታካሚዎች

Participant Survey: Experiences with therapy - Patients

የሕክምና ክፍለ ጊዜዎች ብዛት (Number of treatment sessions)

◻ 1ኛ ክፍለ ጊዜ 1st session ◻ 2ኛ ክፍለ ጊዜ 2nd session ◻ 3ኛ ክፍለ ጊዜ 3rd session ◻ 4ኛ ክፍለ ጊዜ 4th session ◻ 5ኛ ክፍለ ጊዜ 5th session

◻ Other:____

የመጨረሻው የሕክምና ክፍለ ጊዜ (Last treatment session)

◻ 1ሳምንት በፊት 1 week ago (2ሳምንት በፊት 2 weeks ago (ሌላ, ይግለጹ: ________________________________ Other, Specify

◻ አልተተገበረም (የቀድሞ ህክምና የለም) Not applicable (no previous treatment)

ከህክምናው ክፍለ ጊዜ በኋላ በ 20 ደቂቃዎች ውስጥ የሚጠየቁ ጥያቄዎች
Questions to be asked within 20 minutes after treatment session

ለጥያቄዎች መልስ ሰጥተዋል: (Questions answered:)

◻ በተሳታፊ ብቻ By participant alone
◻ በወላጅ / ኃላፊነት የሚሰማው አዋቂ (ልጆች) By a parent / responsible adult (children)
◻ በጓደኛ By a friend
◻ በወላጅ / ኃላፊነት የሚሰማው አዋቂ / ጓደኛ እርዳታ With the *help* of a parent/responsible adult/friend

1) **ዛሬ ስለ ህክምናዎ ፍርሃት ተሰምቷችዋል?** (Did you feel nervous about the treatment today?)

◻ አዎ (Yes) ◻ አይ (No)

2) **ዶክተሩ ሂደቱን አብራርተውልዎታል?** (Did the doctor explain the procedure to you?)

◻ አዎ (Yes) ◻ አይ (No)

3) **ስለ ሂደቱ የዶክተሩን ማብራሪያ ተረድተዋል?** (Did you understand the doctor's explanation about the procedure?)

◻ አዎ (Yes) ◻ አይ (No)

4) **መርፌዎችን ትፈራለህ/ትፈሪያለሽ?** (Are you afraid of needles?)

◻ አዎ (Yes) ◻ አይ (No)

<u>አዎ ከሆነ</u>: የመርፌ ፍራቻህ ከዚህ በፊት ምንም አይነት ህክምና ወይም ክትባት እንዳትወስድ አድርጎሃል?
<u>If yes</u>: Has your fear of needles stopped you from getting any treatments or vaccinations in the past?

◻ አዎ (Yes) ◻ አይ (No)

5) **ከዚህ በፊት በቆዳ ሌይሽማንያሲስ ምክንያት በወሰዱት የመርፌ ህክምና (ኢንትራሊዥናል ቴራፒ) መጥፎ ገጠመኝ አልዎት?**
Did you have bad experiences with injections in skin lesions (*intralesional therapy*) caused by cutaneous leishmaniasis before?

◻ አዎ (Yes) ◻ አይ (No)
◻ አልተተገበረም (የቀድሞ ህክምና የለም) Not applicable (no previous therapy)

<u>አዎ ከሆነ ይግለጹ</u>:
*<u>If **yes**, specify</u>:*

◻ አሉታዊ ተጽኖዎች Adverse effects
◻ አልጠቀመኝም Did not help
◻ ፍርሃት Fear
◻ ሌላ, ይግለጹ: ________________________ Other, specify

አሉታዊ ተፅእኖዎች ካሉ, ይግለጹ: *If **adverse effects**, specify:*

☐ **ህመም, ይግለጹ**:
Pain, *specify:*
☐ የመርፌ-ቦታ ህመም Injection-site pain
☐ የማቃጠል ስሜት Burning sensation
☐ ለመንካት ህመም Pain to touch

☐ የባክቴሪያ ኢንፌክሽን Bacterial infection
☐ ዉሃ ያዘለ እብጠት Blistering
☐ የቀለም ለውጥ Colour change
☐ በመርፌ-ቦታ ላይ እብጠት Swelling at injection-site
☐ ማሳከክ Itching
☐ የአለርጂ Allergic reaction / anaphylaxis
☐ ጠባሳ Scarring
☐ ሌላ, ይግለጹ: ________________________ Other, specify

6) ከዚህ በፊት በቆዳ ሌይሽማንያሲስ ምክንያት በቆዳ ላይ ቀዝቃዛ ነገር (ክሪዮቴራፒ) በሚደረግ ሰኣት መጥፎ ነገር አጋጥመውዎታል?
Did you have bad experiences with application of cold to a skin lesion caused by cutaneous leishmaniasis (*cryotherapy*) before?

☐ አዎ (Yes)
☐ አይ (No)
☐ አልተተገበረም (የቀድሞ ህክምና የለም) Not applicable (no previous therapy)

አዎ ከሆነ ይግለጹ:
*If **yes**, specify*

☐ አሉታዊ ተጽኖዎች Adverse effects
☐ ፍርሃት Fear
☐ አልጠቀመኝም Did not help
☐ ሌላ, ይግለጹ: ______________________ Other, specify

አሉታዊ ተፅእኖዎች ካሉ, ይግለጹ:
*If **adverse effects**, specify*

☐ **ህመም, ይግለጹ**:
***Pain**, specify*
☐ የመርፌ-ቦታ ህመም Injection-site pain
☐ የማቃጠል ስሜት Burning sensation
☐ ለመንካት ህመም Pain to touch

☐ የባክቴሪያ ኢንፌክሽን Bacterial infection
☐ ዉሃ ያዘለ እብጠት Blistering
☐ የቀለም ለውጥ Colour change
☐ በመርፌ-ቦታ ላይ እብጠት Swelling at injection-site
☐ ማሳከክ Itching
☐ የአለርጂ Allergic reaction / anaphylaxis
☐ ጠባሳ Scarring
☐ ሌላ, ይግለጹ: ________________________ Other, specify

7) ዛሬ ያለዎትን ምቾት ማጣት ለመቀነስ የሚረዱ ነገሮች ተጠቅመዋል?
Did you use anything to help reduce the discomfort today?

☐ አዎ (Yes)
☐ አይ (No)

አዎ ከሆነ ይግለጹ: *If **yes**, specify:*

☐ ፓራሲታሞል Paracetamol
☐ ኤን.ኤስ.ኤ.አይ.ዲ (ኢቡፕሮፌን ፣ አስፕሪን ፣ ዳይክሎፌናክ) NSAID (Ibuprofen, Aspirin, Diclofenac)
☐ ቆዳ ላይ ያሚቀባ ማደንዘዣ ክሬም Topical anaesthetic cream

መቼ ነው የተጠቀምከው/ሽዉ?
When did you use it?
☐ ወደ ሆስፒታል ከመድረሴ በፊት Prior to arrival at the hospital
☐ ሆስፒታል ውስጥ At the hospital

8) ዛሬ በህክምና ወቅት ያጋጠመዎት ህመም ከጠበቁት ጋር ሲነጻጸር እንዴት ነበር?
How was the pain you experienced during treatment today compared to what you expected it to be?

- ☐ *በጣም ከተጠበቀው በላይ* — *Much more* than expected
- ☐ *ከተጠበቀው በላይ* — More than expected
- ☐ እንደተጠበቀው — As expected
- ☐ *ከተጠበቀው ያነሰ* — Less than expected
- ☐ *ከተጠበቀው በጣም ያነሰ* — *Much less* than expected

9) ካለፈው የሕክምና ክፍለ ጊዜ ጋር ሲነጻጸር, ዛሬ በሕክምናው ወቅት ያለው ህመም፤
Compared to the previous treatment session, the pain during treatment today was:

- ☐ *የበለጠ ከባድ* — *Much more* severe
- ☐ *ከባድ* — More severe
- ☐ ተመሳሳይ — The same
- ☐ በትንሹ ከባድ — Less severe
- ☐ *በጣም በትንሹ ከባድ* — *Much less* severe
- ☐ አልተተገበረም (የቀድሞ ህክምና የለም) — Not applicable (no previous treatment)

10) ስለሚቀጥለው የሕክምና ክፍለ ጊዜ ምን ያስባሉ?
How do you feel about the next treatment session?

- ☐ ፍራቻ (Nervous)
- ☐ ዘና ያለ ስሜት (Relaxed)
- ☐ ምንም (Neither)

ፍራቻ ከሆነ፡ ለምንድነው የመረበሽ/ፍራቻ ስሜት የሚሰማዎት?

If ***nervous***: Why do you feel nervous?

- ☐ በሕክምና ምክንያት የሚከሰት ህመም በመፍራት — Fear of pain caused by treatment
- ☐ የመታከሚያዉ ወጪ — Costs of being treated
- ☐ ወደ ሆስፒታል መመላለሱ — Having to come back to the hospital
  ይግለጹ፡
  Specify
  - ☐ ሆስፒታል ስለማልወድ (Do not like hospitals)
  - ☐ ከሆስፒታል ያለው ርቀት (Distance to the hospital)
  - ☐ ሌላ, ይግለጹ: ________________
    Other, specify
- ☐ ሌላ, ይግለጹ: ________________________
  Other, specify

11) ካለፈው የሕክምና ክፍለ ጊዜ ጋር ሲነጻጸር, ዛሬ እኔ፤
Compared to the previous treatment session, today I was:

- ☐ *የበለጠ በጣም ፍርሃት* — *Much more* nervous
- ☐ *የበለጠ ፍርሃት* — More nervous
- ☐ ልክ እንደበፊቱ ፍርሃት — As nervous as before
- ☐ ትንሽ ፍርሃት — Less nervous
- ☐ በጣም ትንሽ ፍርሃት — *Much less* nervous
- ☐ አልተተገበረም (የቀድሞ ህክምና የለም) — Not applicable (no previous treatment)

12) ህክምና ከወሰዱ በኋላ የቆዳዎ ችግር ተሻሽሏል?
Has your skin problem improved since you have been having treatment?

- ☐ አዎ (Yes)
- ☐ አይ (No)
- ☐ አልተተገበረም (የቀድሞ ህክምና የለም) — Not applicable (no previous treatment)

Doctor ID:_______________________

Appendix S1 C:
Participant Survey LCL Ethiopia Study: Experiences with LCL Therapy – Doctors (dermatologists)

I) Clinical Experience

1) What is your professional stage?

- ◻ Trainee
- ◻ Attending physician

2) What training did you receive for applying *intralesional therapy* for localised cutaneous leishmaniasis (LCL)?

- ◻ Observation of experienced doctors
- ◻ Applied under supervision of experienced doctor
- ◻ Received didactic teaching (course)
- ◻ Practical training in intralesional therapy (course)

3) What training did you receive for applying *cryotherapy* for LCL patients?

- ◻ Observation of experienced doctors
- ◻ Applied under supervision of experienced doctor
- ◻ Received didactic teaching (course)
- ◻ Practical training in cryotherapy (course)

4) How long have you been administering *intralesional therapy* independently?

- ◻ < 6 months
- ◻ 6-12 months
- ◻ 1-2 years
- ◻ > 2 years

5) How long have you been administering *cryotherapy* independently?

- ◻ < 6 months
- ◻ 6-12 months
- ◻ 1-2 years
- ◻ > 2 years

II) Views on LCL therapy

6) In your experience, how important are the following factors for the risk of discontinuation of *intralesional therapy*?

Low tolerability (e.g. due to pain)

- ◻ Very important
- ◻ Important
- ◻ Moderately important
- ◻ Slightly important
- ◻ Unimportant

Distance to the hospital

- ◻ Very important
- ◻ Important
- ◻ Moderately important
- ◻ Slightly important
- ◻ Unimportant

Costs of being treated

Doctor ID:______________________

- ◻ Very important ◻ Important ◻ Moderately important
- ◻ Slightly important ◻ Unimportant

Beliefs (no trust in modern medicine/in the treatment itself)

- ◻ Very important ◻ Important ◻ Moderately important
- ◻ Slightly important ◻ Unimportant

Lacking understanding of the illness

- ◻ Very important ◻ Important ◻ Moderately important
- ◻ Slightly important ◻ Unimportant

Lacking understanding of the treatment

- ◻ Very important ◻ Important ◻ Moderately important
- ◻ Slightly important ◻ Unimportant

Migration

- ◻ Very important ◻ Important ◻ Moderately important
- ◻ Slightly important ◻ Unimportant

Other factors

- ◻ Specify: ____

7) In your experience, how important are the following factors for the risk of discontinuation of *cryotherapy*?

Low tolerability (e.g. due to pain)

- ◻ Very important ◻ Important ◻ Moderately important
- ◻ Slightly important ◻ Unimportant

Distance to the hospital

- ◻ Very important ◻ Important ◻ Moderately important
- ◻ Slightly important ◻ Unimportant

Costs of being treated

- ◻ Very important ◻ Important ◻ Moderately important
- ◻ Slightly important ◻ Unimportant

Beliefs (no trust in modern medicine/in the treatment itself)

- ◻ Very important ◻ Important ◻ Moderately important
- ◻ Slightly important ◻ Unimportant

Lacking understanding of the illness

- ◻ Very important ◻ Important ◻ Moderately important
- ◻ Slightly important ◻ Unimportant

Lacking understanding of the treatment

- ◻ Very important ◻ Important ◻ Moderately important
- ◻ Slightly important ◻ Unimportant

Migration

- ☐ Very important ☐ Important ☐ Moderately important
- ☐ Slightly important ☐ Unimportant

Other factors

- ☐ Specify: ____

8) In your experience, what factors lead to decreased tolerability of *intralesional therapy*?

Amount of volume injected ☐ Yes ☐ No

Number of separate injections ☐ Yes ☐ No

Previous injections ☐ Yes ☐ No

Age of the patient ☐ Yes ☐ No

*If **yes**, specify which age, in your experience, has the lowest tolerability:*

- ☐ < 5 year olds ☐ 5-10 year olds
- ☐ 11-20 year olds ☐ 21-30 year olds
- ☐ 31-40 year olds ☐ 41-50 year olds
- ☐ 51-60 year olds ☐ >60 year olds

Gender ☐ Yes ☐ No

*If **yes**, specify which gender, in your experience, has the lowest tolerability:*

- ☐ Male ☐ Female

☐ **Other factors** Specify: ____

9) In your experience, what factors lead to decreased tolerability of *cryotherapy*?

Duration of freeze ☐ Yes ☐ No

Number of freeze/thaw cycles ☐ Yes ☐ No

Previous cryotherapy ☐ Yes ☐ No

Age of the patient ☐ Yes ☐ No

*If **yes**, specify which age, in your experience, has the lowest tolerability:*

- ☐ < 5 year olds ☐ 5-10 year olds
- ☐ 11-20 year olds ☐ 21-30 year olds
- ☐ 31-40 year olds ☐ 41-50 year olds
- ☐ 51-60 year olds ☐ >60 year olds

Gender ☐ Yes ☐ No

*If **yes**, specify which gender, in your experience, has the lowest tolerability:*

- ☐ Male ☐ Female

☐ **Other factors** Specify: ____

10) Do you think there is any difference in the tolerability of *combination-therapy (cryotherapy plus intralesional therapy)*, compared to *cryotherapy* or *intralesional therapy* alone?

*Doctor ID:*_______________________

◻ Yes ◻ No

*If **yes**, specify:*

◻ Less painful ◻ More stressful due to longer duration of procedure(s)

◻ Other

*If **other**, specify:* ______________________________

III) Application of treatment

11) How do you decide how many injections a patient needs per session when administering *intralesional therapy*? What does it depend on?

➔ ***Open question; do not read out the answers below, but tick the boxes if item is mentioned by participant***

◻ Size of the lesion ◻ Amount of saturation/swelling of the lesion through intralesional therapy

◻ Prior response to treatment ◻ Other

*If **other**, specify:*

____

12) Do you time how long you are freezing for when applying *cryotherapy*?

◻ Yes ◻ No

*If **yes**: How do you time the freeze?*

➔ ***Open question; do not read out the answers below, but tick the boxes if item is mentioned by participant***

◻ Counting the seconds ◻ Timer (mobile phone/watch)

◻ Other, specify: ____
