# Supplementary material for: Experiences of Individuals with Cutaneous Leishmaniasis Receiving Intralesional Sodium Stibogluconate or Liquid Nitrogen Cryotherapy in Addis Ababa, Ethiopia—A Cross-Sectional Study
Source: Trop Med Infect Dis. 2025 Jul 23;10(8):203. doi: 10.3390/tropicalmed10080203 (PMC12390606; doi:10.3390/tropicalmed10080203)
Supplement: Supplementary file 1 [file tropicalmed-10-00203-s001.zip › File S2_STROBE Checklist.pdf]

STROBE Statement—Checklist of items that should be included in reports of ***cross-sectional studies***

	Item No	Recommendation
**Title and abstract**	1	(*a*) Indicate the study's design with a commonly used term in the title or the abstract
		(*b*) Provide in the abstract an informative and balanced summary of what was done and what was found<br>Included in the title and on p. 1
**Introduction**		
Background/rationale	2	Explain the scientific background and rationale for the investigation being reported<br>Included on p. 2
Objectives	3	State specific objectives, including any prespecified hypotheses<br>Included on p. 2
**Methods**		
Study design	4	Present key elements of study design early in the paper<br>Included on p. 3
Setting	5	Describe the setting, locations, and relevant dates, including periods of recruitment, exposure, follow-up, and data collection<br>Included on pp. 2-3
Participants	6	(*a*) Give the eligibility criteria, and the sources and methods of selection of participants<br>Included on p. 3
Variables	7	Clearly define all outcomes, exposures, predictors, potential confounders, and effect modifiers. Give diagnostic criteria, if applicable<br>NA
Data sources/ measurement	8*	For each variable of interest, give sources of data and details of methods of assessment (measurement). Describe comparability of assessment methods if there is more than one group<br>Included on p. 3
Bias	9	Describe any efforts to address potential sources of bias<br>Included on p. 3
Study size	10	Explain how the study size was arrived at<br>Included on p. 3
Quantitative variables	11	Explain how quantitative variables were handled in the analyses. If applicable, describe which groupings were chosen and why<br>Included on p. 3
Statistical methods	12	(*a*) Describe all statistical methods, including those used to control for confounding
		(*b*) Describe any methods used to examine subgroups and interactions
		(*c*) Explain how missing data were addressed
		(*d*) If applicable, describe analytical methods taking account of sampling strategy
		(*e*) Describe any sensitivity analyses<br>Included on p. 3
**Results**		
Participants	13*	(a) Report numbers of individuals at each stage of study—eg numbers potentially eligible, examined for eligibility, confirmed eligible, included in the study, completing follow-up, and analysed
		(b) Give reasons for non-participation at each stage
		(c) Consider use of a flow diagram

		Included on p. 4
Descriptive data	14*	(a) Give characteristics of study participants (eg demographic, clinical, social) and information on exposures and potential confounders
		(b) Indicate number of participants with missing data for each variable of interest<br>Included on pp. 5-7
Outcome data	15*	Report numbers of outcome events or summary measures<br>Included on pp. 5-10
Main results	16	(*a*) Give unadjusted estimates and, if applicable, confounder-adjusted estimates and their precision (eg, 95% confidence interval). Make clear which confounders were adjusted for and why they were included
		(*b*) Report category boundaries when continuous variables were categorized
		(*c*) If relevant, consider translating estimates of relative risk into absolute risk for a meaningful time period<br>Included on p. 7 and in S1 Table
Other analyses	17	Report other analyses done—eg analyses of subgroups and interactions, and sensitivity analyses<br>NA
**Discussion**		
Key results	18	Summarise key results with reference to study objectives<br>Included on pp. 10-11
Limitations	19	Discuss limitations of the study, taking into account sources of potential bias or imprecision. Discuss both direction and magnitude of any potential bias<br>Included on p. 11
Interpretation	20	Give a cautious overall interpretation of results considering objectives, limitations, multiplicity of analyses, results from similar studies, and other relevant evidence<br>Included on p. 11
Generalisability	21	Discuss the generalisability (external validity) of the study results<br>Included on p. 11
**Other information**		
Funding	22	Give the source of funding and the role of the funders for the present study and, if applicable, for the original study on which the present article is based<br>Included on p. 12

*Give information separately for exposed and unexposed groups.

Note: An Explanation and Elaboration article discusses each checklist item and gives methodological background and published examples of transparent reporting. The STROBE checklist is best used in conjunction with this article (freely available on the Web sites of PLoS Medicine at http://www.plosmedicine.org/, Annals of Internal Medicine at http://www.annals.org/, and Epidemiology at http://www.epidem.com/). Information on the STROBE Initiative is available at www.strobe-statement.org.
