# Supplementary material for: Experiences of Individuals with Cutaneous Leishmaniasis Receiving Intralesional Sodium Stibogluconate or Liquid Nitrogen Cryotherapy in Addis Ababa, Ethiopia—A Cross-Sectional Study
Source: Trop Med Infect Dis. 2025 Jul 23;10(8):203. doi: 10.3390/tropicalmed10080203 (PMC12390606; doi:10.3390/tropicalmed10080203)
Supplement: Supplementary file 1 [file tropicalmed-10-00203-s001.zip › tropicalmed-3625878-TabS1.pdf]

Table S1. Risk of Wong-Baker FACES pain scores ≥8 with individual and healthcare-related factors, n = 35.

Variable	Subgroups	No. of individuals (%)	No. with pain score ≥8 (%)	Crude odds ratio	95% confidence interval
**Individual factors**					
Age	8-15 years	5 (14.3)	4 (80)	1.0	1.0
	≥16 years	30 (85.7)	14 (46.7)	0.22	0.02-2.43
Sex	Male	22 (62.9)	16 (72.7)	1.0	1.0
	Female	13 (37.1)	8 (61.5)	0.43	0.10-1.85
Educational level	No formal education/Primary	18 (51.4)	10 (55.6)	1.0	1.0
	Secondary/ Post-secondary	17 (48.6)	8 (47.1)	0.71	0.18-2.76
Location of index lesion	Facial lesion	24 (68.6)	17 (70.8)	1.0	1.0
	Other	11 (31.4)	7 (63.6)	1.2	0.28-5.13
Size of index lesion	<40 mm	18 (51.4)	14 (77.8)	1.0	1.0
	≥40 mm	17 (48.6)	10 (58.8)	1.13	0.29-4.33
Duration of index lesion	<12 months	14 (40)	9 (64.3)	1.0	1.0
	≥12 months	21 (60)	15 (71.4)	1.78	0.44-7.2
**Healthcare-related factors**					
Type of treatment	Intralesional SSG	3 (8.6)	2 (66.7)	1.0	1.0
	Cryotherapy	13 (37.1)	7 (53.9)	0.60	0.04-10.13
	Combination therapy	19 (54.3)	15 (79)	5.60	0.34-91.76
Treatment session number	1st-5th	20 (55.6)	12 (60)	1.0	1.0
	≥6th	15 (44.4)	6 (40)	0.44	0.11-1.83
Training level of resident	1st year	8 (22.9)	7 (87.5)	1.0	1.0
	2nd or 3rd year	27 (77.1)	17 (63)	0.27	0.04-1.71
**For participants receiving cryotherapy (n=32)**					
Type of application	Cotton bud	14 (43.8)	7 (50)	1.0	1.0
	Cryotherapy spray device	18 (56.3)	15 (83.3)	6.5	1.13-37.41
Number of freeze cycles	1	11 (34.4)	6 (54.6)	1.0	1.0
	≥2	21 (65.6)	11 (52.4)	0.92	0.21-4.06
Total duration of the freezes	15-45 sec	16 (50)	10 (62.5)	1.0	1.0
	≥46 sec	16 (50)	7 (43.8)	0.47	0.11-2.02
**For participants receiving intralesional SSG (n=22)**					
Number of separate injections	1-5	12 (54.5)	10 (83.3)	1.0	1.0
	≥6	10 (45.5)	5 (50)	0.2	0.02-1.71
Amount of SSG injected	0.1-0.4 ml	14 (63.6)	10 (71.4)	1.0	1.0
	0.5-0.8 ml	8 (36.4)	7 (87.5)	1.67	0.23-12.27

No., number; SSG, sodium stibogluconate.
